# Anæsthesia, Local and General

**Published:** 1896-07

**Authors:** A. C. Hewitt

**Affiliations:** Chicago, Ill.


THE
AMERICAN JOURNAL
—OF—
DENTAL SCIENCE.

VOL. XXX. BALTIMORE, JULY, 1896. NO. 3.

ARTICLE I.

### ANÆSTHESIA, LOCAL AND GENERAL.

BY A. C. HEWITT, M. D., CHICAGO, ILL.

Read before the Iowa State Dental Society.

(Continued from last Number.)

### CHLOROFORM.

Of this agent some of you have heard me speak before; to such, what I feel in duty bound to say will be largely a repetition. No one doubts its energy as a general anesthetic; no well informed surgeon, physician or dentist will doubt its terrible toxic vigor.

Close study of its effects and an extended experimental investigation of it, commenced in the boyhood of my profession, continued up to the present time, at first prosecuted under difficulties so appalling to recollection that I now wonder at my courage and persistence, difficulties chiefly due to my lack of experience. Unaided by definite literature on the subject, fiercely opposed by physicians and accoucheurs, when I began its use in aid of parturition, assailed as a reckless and "dare devil" experimentalist, stared at by what there was then of a dental profession (?) and prophesied evil of for my pertinacity, I was early impressed with its treasure of merciful benefaction, only waiting to be worked out from its arcanum, and to be intelligently interposed between the forceps, bistoury, catling and shrinking, quivering human nerves. Would that my researches had been aided by a larger intelligence and that the words I now utter might reach the ears of a far greater audience than is before me, laden with the spirit of profound assurance I am compelled to bear, saturated and sent home by all the claimed force of hypnotic suggestion.

Since the time when, alone in my office in Michigan on a bright Sabbath morning, I extracted my right inferior second molar, using both hands on the forceps, when under the analgic or obtundent influence of chloroform, absolutely without pain or shock (but filled the while with dread), I have known that lethea and death not only were hidden in chloroform's fumes, but that the wings of an angel of mercy were poised in its vapor, ready at call to sweep into temporary sleep the ultimate nervules of sensation without shadowing into inertia the breathing and heart beat necessary to life's continuance.

It is well for me and for my personal freedom from prison walls, or worse, that the analgic, obtundent forces of chloroform were, and are, as harmless as they are proven. I had no guide in my experiments, while developing the, to me, new discovery; not a hint in the pharmaceutic, medical or surgical literature at my command did I have to aid me. On the contrary, "shock under partial anesthesia" was early recorded, as it is still believed by many, mistaking shock of chloroform to the peripheral nerves spread upon the mouth, nose, throat and lungs, and its consequences, for the shock of forcep or knife.

It is difficult for you to imagine the fierceness of opposition I encountered in the years of my medical and surgical practice on account of my persistence in giving chloroform for minor operations in surgery, without calling other members of the profession in counsel. If a felon was to be lanced, a carbuncle to be carved crucially. a phlegmon to be lanced, I gave chloroform in the same way that I do now, regardless of what professional men said, so long as my patients asked it and were satisfied with results.

If a woman in labor chose to avail herself of the analgic use of chloroform, I gave it; at first with fear, I confess, but soon with great satisfaction. From that early date to the time I now write, I have thus administered it thousands of times to old and young, to hale people and sickly, to nervous and stolid, and it is with pride and reverent gratitude that not a single casuality has marred my advance; not a collapse to affright me, but successes crowning one and another, till my faith in it is a verity, and my admiration for it unbounded. For the last twelve years, in my office at No. 491 W. Adams street, Chicago, I have almost daily, and often many times a day, thus administered chloroform, and not a whisper has gone out of a casuality or attending untoward symptom.

On the contrary, thousands in that time have experienced its beneficence and warmly praised it.

I have thus spoken freely of myself, with seeming egotism of accomplishment, not because of conceit—far from it; but I have no other authority to quote.

So far as I know, I alone have pursued this line of investigation; so far as I am aware, my paper above referred to was the first published in modern times, announcing the discovery and the mode of administration; I believe my voice alone has sounded a challenge to the proposition that "There is danger from shock while operating upon a person partially anesthetized." I now repeat the challenge. If the statement is true, that since that Sabbath morning spoken of no casualties have resulted from operating under partial anesthesia, may we not conclude that *said* "shock" is but a *bogy*, a *myth*.

I am firm in the conviction that as a general anesthetic for the temporary suppression of nervous sensation, chloroform stands first, as it certainly does for lethal effect in prolonged operations. It only needs intelligence and caution of a high order in its administration to render it safe. Dr. Syme used it in five thousand cases with perfect safety, and I doubt not that had he used it in thrice or ten times that number, the result would have been the same had he not relaxed in prescient care. Dr. Sayre gave it in several thousand cases safely. It were a violent supposition to claim that the nearly ten thousand cases in the hands of those distinguished surgeons were all exceptionally safe ones. I dwell on this for the purpose of adding force to what I shall presently say as to the safety of partial anesthesia for dental uses.

In passing, I wish to enter a vigorous protest against a practice almost universal among general surgeons, and one followed too often by oral surgeons; namely, that of giving the administration of anesthetics for prolonged operations into the hands of incompetent persons, generally that of students or internes, often of medical friends invited to witness the operation.

The best anesthetic teachings have come from the dental profession. The surgical world may, without loss of dignity, listen to even my warning. Deaths from chloroform narcosis need not and *should not* occur.

### How to Avoid the Danger.

*First.* Prepare the patient by giving a respiratory and cardiac stimulant, and one ounce of pure brandy with half a glass of water should be given, or its equivalent in some other alcoholic stimulant; a hypodermic injection of 1-120 of a grain of sulphate of strophanthin and 1-60 of a grain of sulphate of atropin should be given, or, what is more convenient, place 1-60 of a grain of the former and 1-50 of a grain of the latter well back upon the tongue, allowing it slowly to dissolve.

Commence inhalations of chloroform well mixed with atmospheric air in such quantities as will not induce flushing of the face, coughing, or the slightest irritation of the nerves of the nose, throat and lungs. Upon the strict observance of this rule depends the avoidance of the first danger to chloroform narcosis. If a mask is used, it should at first be held well away from the face, and passed to and fro, or rooted over the patient's mouth and nose, that the four-times-heavier-than-air vapor shall be well mixed with the atmosphere; by all means do not irritate the nerve sentinels in the nose; mouth and throat, placed there, no doubt, with the especial design of sending irritation to the trigemini and medulla oblongata in presence of dangerous fumes or vapors. I cannot emphasize too strongly the importance of observing these directions.

Thence on, give the chloroform vapor well charged with air, as rapidly as possible without irritation, never minding the pulse, caring nothing for the pupils; leave the heart to itself, but closely observe the breathing, if in the least retarded or shallow, wait a moment or two until regular respiration is established; thence on, keep well within this line. After the first danger of shock is passed, the breathing will hang out the first red light signal of danger. Watch for this signal carefully, and if the operation is long-continued, and respiration becomes lazy, a hypodermic injection of some diffusible stimulant should be given. In the second stage narcosis is generally carried much too far; the slight underlying muscular contractions should in no case be entirely subdued. Tbe muscles of the slaughtered ox, when disemboweled, flayed, beheaded and quartered, will for hours show these contractions when pricked or cut.

It were well for some of our surgeons to go to the slaughter-house and take lessons. As to positions, I prefer the semi-prone or recumbent sitting, if the operation will allow. If the recumbent position is required, place the head in that position well forward, always assumed by a person in strangling; in a position where the gravitation of the tongue will not be down the throat; no person will "swallow his tongue" if the head is in the proper position. If the respiration is properly watched and guarded, no forceps will be required for dragging forward the tongue.

The operator or oral surgeon should in no case be required to pay the least attention to the anesthetic, but be left as free to work as coolly and leisurely as though his subject were moribund, a condition into which too many of them pass by reason of the non-observance of the cautions herein given. I hope to see the time when in every college, dental as well as medical or surgical, a chair of anesthesia shall be established at which a complete anesthetic education may be obtained. Would that the progressive and all-alive State of Iowa might lead in this important matter.

### PARTIAL ANESTHETICS.

All authors treating of anesthetics recognize the condition of partial in contradistinction to general anesthesia, and with one accord also treat it as a condition of extreme dangei for painful operations, and especially so in the region of the fifth pair of nerves. How this belief obtained I am utterly at a loss to explain; that I do not accede in the slightest degree to that belief I have stated before.

I would not allude to it again, only that I may the more thoroughly emphasize the caution I have given before, and now repeat, against shock in the early stages of anesthesia. Shock may be defined, sudden depression of vital power passing into reaction or into fatal sinking. This is primary. A secondary shock may occur, which follows after the primary one has passed away. It is this secondary shock alone that may have led to the error, for error I hold it. I have proven it by experiments upon animals, producing partial anesthesia and then inflicting extensive and shocking wounds and bruises, till almost sick at sight of my seeming cruelty, and if the knife or blows did not reach a vital part, so far were they from producing shock; the subjects seemed to know or care very little for them, until quite a time after recovery from the anesthesia.

The major part and the most convincing of my experiments were upon human beings, and in parts supplied by the fifth pair of nerves, so that if shock were possible, I should have, sometime, somewhere, encountered it. I repeat it is a *bogy*, a *myth*. Do not let it awe you. On a former occasion, when treating of this subject, I gave what I thought plain directions as to the mode of giving anesthetics to the point of obtundure to ultimate nervules of sensation, for with them we have to deal in "painless dentistry." (I use the last phrase in a restricted sense, not absolute.) I have been asked to repeat them and especially point out the indications that cry "Enough." Perhaps I can best do so by giving directions how to extract a tooth painlessly, safely and speedily; time, not more than five minutes. Position, sitting in the chair, titled a little back, perhaps twenty degrees from perpendicular; head in convenient position for operator. Paint the gums around with com. cocaine pigment (which I greatly prefer) or with a 4 per cent. solution mur. cocaine, guarded as in foregoing formula, using a small paint brush, as No. 2, with a stippling motion, dabbing the bristles against the tooth toward the free margin of gingivæ. If a little bleeding occurs, so much the better; immediately after commence giving chloroform *purification* from a wide-mouthed bottle (my choice), directing deep, long, steady inhalations across the bottle's mouth, through one nostril, the other being compressed by the operator, held at such a distance below the nose that but a faint odor of the drug can be detected by the patient; at the close of each inhalation, remove the bottle from the course of the exhaled air and immediately replace under the open nostril, for a second inhalation, a trifle nearer each time; rate of breathing, about ten per minute. Operator should keep up a running fire of words to patient, such as "Don't talk," "Steady," Take deep breaths," "Not too fast," "Deeper," "Longer," "Don't forget to breathe," and the like, all the time nearing the bottle to the nose, till at last the air will cross the bottle's mouth almost in a whistle. At this latter time, between each breath, shake the bottle to hasten evaporation of the drug. When sufficient is given, generally the first symptom apparent is a slightly lazy, tremulous, drooping, slow winking motion of the eyelids, and at the same time a slight relaxation of muscles and a settling down in the chair; often now the ala of the free nostril will partially close down upon the septum of the nose, so as to somewhat obstruct inhalation. Watching closely the breathing, you will see a shallower inhibition with lengthening intervals. Now say to your patient, "Breathe" (in a sharp tone of command), "Breathe deeper, longer," "Don't forget to breathe." (To yourself, "Keep cool, firm, collected.") Repeat these words to your patient, at the same time giving a sharp justle of the body; about this time there will be a distinct pause in respiration. notwithstanding your command and shake. Do not be in the least alarmed. There is no pallor, no cyanosis, spasms or excitement, simply an interim in the inhalations.

Now is your time, but do not hurry, deliberately put down your bottle, take up your forceps carefully, leisurely adjust the blades and extract the tooth. As you close down on the tooth and begin to pull, your patient will breathe, frequently start up with a moan, soon to settle back and seem to say, "Pull away, I don't care," and after the tooth is out, remain with head back and mouth open, long enough for the extraction of several others, and till he is aroused and told to lean over and free the mouth of blood, which will be done, followed by a rapid restoration and profuse expressions of delight in your method and skill.

Precisely in the same way may partial anesthesia be produced for the relief from the acute pain of any dental burring, drilling, lancing, scraping, grinding or prodding.

The terror of minor surgical operations may thus be defied, *and it is safe* not only in my hands, but in yours, *of* two rules of caution are observed:

*First.* Do not give shock to the peripheral nerves of the nose, mouth, throat and lungs, so closely contiguous to the trigemini and medulla oblongata. Why? Because the medulla contains the cardiac and respiratory centers and the nuclei of the hypoglossal spinal accessory and glosso-pharyngeal nerve. As well a stroke with a sand-bag.

*Second.* Do not, after having passed the first danger, carry the anesthesia so far as to put to sleep the ganglia controlling respiration, heart beat and peristalsis.

It were criminal to do either. Can I give a more emphatic caution?

### A WORD TO MY CRITICS.

At Davenport, in the joint conventions of Iowa and Illinois, at the Columbian Dental Congress and at Old Point American Association, last summer, the trend of criticism was the same. In brief it was, "The numerous deaths caused by chloroform proved it unsafe." And that, though it might be safe in my hands, it was not advisable that dentists in general should use it, even for partial anesthesia. To the first I reply, admitting numerous deaths, more numerous than they claim, but insist that it is because administered improperly; in other words, the dosage is improper. As well argue that because two grains of strychnine is a fatal dose, that therefore one sixteenth of a grain must not be given. Because an overdose of morphine is dangerous, therefore an eighth or fourth of a grain should not be given. I grant and urge that for full anesthesia, for prolonged operations chloroform is dangerous, and so are ether, pental and all the rest, and should be given for that purpose only by those especially educated for that work and trained by experience to avoid the dangers *always incident to unconsciousness* of anesthetization. Such administration of any anesthetic may be likened to the necessity of giving the largest possible safe dose of strychnine, even to the vergo of imminent danger, for the cure of paralysis, which should never be attempted except by those of undoubted skill and care.

An interne, or nurse, may be instructed and easily trained to administer 1-60, 1-40, or 1-15 of a grain of that drug, and that with absolute safety, even taking idiosyncrasies into account, but to resort to the larger dose is quite another matter.

To insist that partial anesthesia cannot be safely produced by any or all of the members of the dental profession, aided by directions that I have given, but that the same is safe in my hands, is a compliment as unmerited by myself as it is doubtful justice to those who are educated thoughtful men, so certified by diploma and license.

The work that I entered upon in the adolescence of my professional life, and without casualty have prosecuted until the present, is not one of danger if the guides I have given are followed.—*The Dental Review.*

